# Assessment of immunosuppressive activity of human mesenchymal stem cells using murine antigen specific CD4 and CD8 T cells *in vitro*

**DOI:** 10.1186/scrt339

**Published:** 2013-10-22

**Authors:** Cristina Nazarov, Jessica Lo Surdo, Steven R Bauer, Cheng-Hong Wei

**Affiliations:** 1 Gene Transfer and Immunogenicity Branch, Division of Cellular and Gene Therapies, Office of Cellular, Tissue, and Gene Therapies, FDA, Center for Biologics Evaluation and Research, Bethesda, MD, USA; 2 Cellular and Tissue Therapies Branch, Division of Cellular and Gene Therapies, Office of Cellular, Tissue, and Gene Therapies, FDA, Center for Biologics Evaluation and Research, Bethesda, MD, USA; 3 FDA/Center for Biologics Evaluation and Research, NIH Bldg 29B 1NN10 HFM-725, 8800 Rockville Pike, Bethesda, MD 20892, USA

## Abstract

**Introduction:**

Mesenchymal stem cells (MSCs) have immunosuppressive activity. They do not induce allospecific T cell responses, making them promising tools for reducing the severity of graft versus host disease (GVHD) as well as treating various immune diseases. Currently, there is a need in the MSC field to develop a robust *in vitro* bioassay which can characterize the immunosuppressive function of MSCs.

**Methods:**

Murine clonal CD4 and CD8 T cells were stimulated with cognate peptide antigen and antigen presenting cells (APCs) in the absence or presence of human MSCs, different aspects of T cell activation were monitored and analyzed using flow cytometery, real time RT-PCR and cytokine measurement.

**Results:**

Human MSCs (hMSCs) can alter multiple aspects of murine T cell activation induced by stimulation with specific antigen, including: reduced proliferation, inhibited or stimulated cell surface marker expression (CD25, CD69, CD44 and CD62L), inhibited mRNA expression of transcription factors (T-bet and GATA-3) and decreased cytokine expression (interferon-gamma, interleukin-10). Disappearance of activation-induced cluster formation and decreased apoptosis of CD8 T cells were also observed. Moreover, the effects are specific to MSCs; incubating the T cells with non-MSC control cell lines had no effect on T cell proliferation and activation.

**Conclusions:**

Clonal murine T cells can be used to measure, characterize, and quantify the *in vitro* immunosuppressive activity of human MSCs, representing a promising approach to improve bioassays for immunosuppression.

## Introduction

Mesenchymal stem cells (MSCs) are mesoderm-derived cells that are found in virtually all tissues and function as precursors of non-hematopoietic connective tissues with the capacity to differentiate into mesenchymal and non-mesenchymal cell lineages. They are the precursors of three main cell types of the mesodermal lineage, including osteocytes, chondrocytes and adipocytes [1-3]. These cells are commonly described as positive for CD73, CD105 and CD90 and negative for hematopoietic (CD45) and vascular (CD31) markers [4]. Their properties have been extensively studied in recent years. Since MSCs are capable of differentiating into several cell lineages [5], they have been used in investigational studies to treat a variety of tissue injuries both in experimental and clinical settings [6-8].

An interesting aspect of MSCs is the finding that they exert immunoregulatory activities. MSCs from various species (human, rodents and primates) can suppress the T cell response to mitogenic and polyclonal stimuli [9,10] and to specific peptide antigens [11]. MSCs have a similar effect on both memory and naïve T cells [12], as well as both CD4^+^ and CD8^+^ subsets [13]. The immunosuppressive effects of MSCs make them attractive candidates for a variety of cellular therapies, including treatment of immune disorders.

MSCs express low levels of MHC I and do not express MHC II or co-stimulatory molecules; they are, therefore, considered to be immune privileged cells and can be successfully transplanted across allogeneic barriers [14]. In addition, large amounts of MSCs can potentially be generated from healthy donors. These unique properties have promoted wide application of MSCs in clinical trials to treat various immune diseases, including multiple sclerosis, Crohn’s disease, type 1 diabetes, systemic lupus erythematosus (SLE) and acute and chronic graft versus host disease (GVHD) [15,16]. Mouse models have been used to test the efficacy for the treatment of GVHD, neurological and systemic autoimmune diseases, sepsis, and acute renal and lung injury, as well as other pathological conditions [17].

Due to the low frequency of MSCs in the bone marrow and the potential for allogeneic therapy, MSCs need to be extensively expanded and passaged to obtain sufficient cell numbers for cell therapies. Therefore, there is a need to understand the role of cell expansion, cell passaging, and donor differences on MSC immunosuppressive capacity. Currently, there are no robust quantitative bioassays suitable for measuring differences in immune-inhibitory activity of MSCs from different donors or at different passages, or under different conditions in large-scale tissue culture expansion. There is a related scientific need to identify the molecular mechanisms underlying MSC-mediated immunosuppression, which also requires accurate assays to measure the immunosuppressive activity of MSCs. Such methods could potentially be used to assess MSCs preparations from various donors and expansion methods or to predict MSC behavior after transplantation.

To address these issues, we developed novel immune inhibition assays using clonal murine T cell populations responding to known peptide antigens, and MSCs derived from human donors. MSCs are known to be immunosuppressive across xenogeneic barriers [18,19], allowing us to assess the use of easily obtained clonal murine T-cells as a method to reduce variability in T-cell based *in vitro* immune suppression assays. Using this system we assessed the immunosuppressive activity of human bone marrow-derived MSCs (hMSCs) on antigen specific, clonal murine T cells. In our system, hMSCs clearly show dose-dependent inhibitory properties, affecting both the proliferation and the activation of antigen specific T cells. We also were able to use this system to investigate some of the molecular mechanisms that participate in cross-species immunosuppression, which may potentially shed light on allogeneic immunosuppressive activities of hMSCs.

## Methods

### Ethics statement

All animal protocols and procedures were approved by the Institutional Animal Care and Use Committees at the Center for Biologics Evaluation and Research (CBER; Protocol #2011-15) and in animal facilities accredited by the Association for Assessment and Accreditation of Laboratory Animal Care International. All experiments were performed according to institutional guidelines.

### Cell culture

hMSCs were purchased (AllCells, Emeryville, CA, USA) at passage one. According to the manufacturer’s and our characterizations, MSCs were negative for hemaotopoietic lineage markers, including CD45, CD34, CD14, CD79α, CD117 and HLA-DR. MSCs were plated in T175 flasks (Greiner Bio-One, Monroe, NC, USA) at 60 cells/cm^2^, expanded in α-MEM (Invitrogen, Carlsbad, CA, USA) supplemented with 16.5% fetal bovine serum (FBS) (JMBioscience, San Diego, CA, USA), Pen Strep and L-glutamine (Invitrogen), and cultured at 37°C and 5% CO_2_. At 80% confluence, 0.25% Trypsin/EDTA (Invitrogen) was used to harvest MSCs. Cells were washed, then cryopreserved in freezing medium containing 5% Dimethyl sulfoxide (DMSO) (Sigma-Aldrich, St. Louis, MO, SA), 30% FBS, 1% penicillin (100 units/ml) and streptomycin (100 μg/ml) (Invitrogen) at 1 × 10^6^ cells/ml at passage three (P3). hMSCs at P3 were thawed, cultured to 80% confluence and harvested for experiments.

The HT-1080 fibrosarcoma cell line was purchased from American Type Culture Collection (ATCC, Manassas, VA, USA). The cell line was plated in T175 flasks, expanded in RPMI-1640 medium supplemented with 10% FBS, Pen Strep and L-glutamine, and cultured at 37°C and 5% CO_2_. Cells at P3 were harvested with Trypsin/EDTA and used in experiments.

### Flow cytometry analysis

hMSCs were incubated with 2.4G2 antibody at 4°C for 30 minutes to block non-specific binding. Primary antibodies used were purchased conjugated to their respective fluorochromes (phycoerythrin (PE); allophycocyanin (APC); fluorescein isothiocyanate (FITC)): anti-CD29-APC, anti-CD44-APC, anti-CD90-FITC, anti-CD73-PE (BD Biosciences, San Jose, CA, USA), anti-CD166-FITC (US Biological, Salem, MA, USA), anti-CD105-PE (Beckman Coulter, Brea, CA, USA) and anti-STRO-1-Alexa647 (Biolegend, San Diego, CA, USA). MSCs were also analyzed for negative markers, including anti-CD34-PE, anti-CD45-PE-Cy7, anti-CD14-ECD, anti-CD79α-PE-Cy5, anti-CD117-APC (Beckman Coulter) and anti-HLA-DR-FITC (BD Biosciences). hMSCs were incubated with antibody at optimized concentrations for 30 minutes at 4°C. Samples were centrifuged, washed twice with phosphate-buffered saline (PBS)/1% fetal bovine serum/0.2% sodium azide, and analyzed in single color using FACSCalibur (Becton Dickinson) or FACS Canto flow cytometers. For flow cytometry analysis of T cells, anti-CD4, anti-CD8, anti-T cell receptor (TCR) Vβ4, anti-TCR Vβ8.1/8.2, anti-CD25, anti-CD69, anti-CD62L, anti-CD44 in various fluorochrome combinations were purchased from BD Biosciences. For all surface marker analysis, TCR transgenic CD4^+^ T cells (BDC2.5 T cells) were first gated using CD4 and TCR Vβ4 double staining, while CD8^+^ TCR transgenic CD8 T cells (8.3 T cells) were gated using CD8 and TCR Vβ8.1/8.2 double staining. Annexin V/7 AAD apoptosis detection kit was purchased from BD Biosciences. CFSE (carboxyfluorescein diacetate, succinimidyl ester) was purchased from Invitrogen (Carlsbad, CA) and was used according to the manufacturer’s instructions.

### Mice

NOD/ShiLtj mice, Nonobese diabetic 8.3 TCR transgenic mice (NOD 8.3) and Nonobese diabetic BDC2.5 TCR transgenic mice (NOD BDC2.5) were purchased from Jackson Laboratories (Bar Harbor, ME, USA) and were maintained in specific pathogen-free conditions according to the guidelines of CBER’s Institutional Animal Care and Use Committee (IACUC). The BDC2.5 TCR transgenic CD4^+^ T cells specifically recognize the I-Ag7 restricted epitope derived from an islet antigen [20], and the 8.3 TCR transgenic CD8^+^ T cells specifically recognize the K^d^-restricted IGRP^206-214^ epitope derived from the islet antigen IGRP (islet-specific glucose-6-phosphatase catalytic subunit-related protein) [21]. These studies were approved by the IACUC of CBER.

### Immunosuppression assay

Spleens and lymph nodes were isolated from NOD 8.3 and NOD BDC 2.5 TCR transgenic mice, then CD4 and CD8 T cells were negatively selected and purified using the mouse CD4^+^ T cell isolation kit and CD8^+^ T cell isolation kit, respectively (Miltenyi Biotec, Auburn, CA, USA). T cells were added to 24-well plates (Becton Dickinson Labware, Franklin Lakes, NJ, USA) (2 × 10^6^ cells/well). Total splenocytes from NOD/ShiLtj mice were irradiated at 4,000 rads and added to the culture as antigen-presenting cells (APCs) (4 × 10^6^ cells/well). In all the experiments APCs are irradiated. Islet-specific glucose-6-phosphatase catalytic subunit–related protein (IGRP^206-214^, VYLKTNVFL) and BDC 2.5 peptides (RVRPLWVRME) were synthesized by the FDA FBR (Facility for Biotechnology Resources) core facility. Peptides were added to a concentration of 1 μg/ml per well. Next, human MSCs were trypsinized, washed and added to the wells at different T cell: MSC ratios. Ratios of 10:1 and 5:1 were found to be effective for our conditions and were used in all experiments. Cells were kept in RPMI 1640 complete medium (containing 10% FBS) in a 37°C incubator for three days after which murine T cells were harvested and analyzed.

For the immunosuppression assay using a transwell setup, hMSCs were cultured on the top level of the HTS Transwell®-24 Well plate with 0.4 μm pores (Corning, Lowell, MA USA) and the T cells together with the irradiated APCs and peptide were cultured in the bottom wells in the same ratios as described above. The cells were grown for three days at 37°C after which they were harvested and analyzed.

### Cytokine analysis

Supernatants were collected at Day 3 of cell culture and stored at -80°C for further analysis. Cytokine concentration was measured using the Mouse Th1/Th2 6-plex Panel kit from Invitrogen according to the manufacturer’s instructions. Samples were acquired and analyzed using a Bio Plex 200 instrument (BioRad, Hercules, CA, USA).

### Real time RT-PCR

Total RNA was extracted from suspension T cells using Pure Link Micro-to Midi RNA extraction kit (Invitrogen), quantified using a Nanodrop 1000 spectrophotometer (Thermo Scientific, Asheville, NC, USA) and stored at -80°C for further analysis. RNA integrity was assessed using an Agilent 2100 Bioanalyzer (Agilent Technologies, Santa Clara, CA, USA) and the RIN (RNA Integrity Number) values were all greater than 9.20. Taqman RT-PCR probes for murine transcription factors T-bet and GATA-3 were purchased (from customized probes) from Applied Biosystems (Foster City, CA). A total of 200 ng of RNA was reverse transcribed into cDNA using a High capacity cDNA Reverse Transcription kit from Applied Biosystems. cDNA was specifically amplified using the ABI 7900 instrument from Applied Biosystems. 18S rRNA was used as endogenous control in all samples. Results were analyzed using SDS 2.3 software (Applied Biosystems).

### Statistical analysis

Data were analyzed using GraphPad Prism 5 software (GraphPad, La Jolla, CA, USA), and Student’s *t*-test was used to compare differences between samples and groups. The differences were considered statistically significant when *P*-value was below 0.05.

## Results

### Human mesenchymal stem cells show typical cellular and functional phenotype

The hMSC line used in this study, PCBM 1632, demonstrated tri-lineage differentiation toward adipo-, osteo- and chondro-genic lineages when using standard induction protocols (Miltenyi-Biotech) (data not shown). The MSC line expressed markers typical of hMSCs [22]. Passage 3 hMSCs show high positive expression for MSC surface markers: CD29 (93.6%), CD44 (98.4%), CD166 (98.3%), CD90 (93%), CD73 (99.6%) and CD105 (98.6%). A small subset of hMSCs (16.9%) was positive for STRO-1, a marker thought to be associated with a clonogenic and more immunosuppressive subpopulation of MSCs [23].

### hMSCs inhibit the proliferation of murine T cells

**Figure 1:**
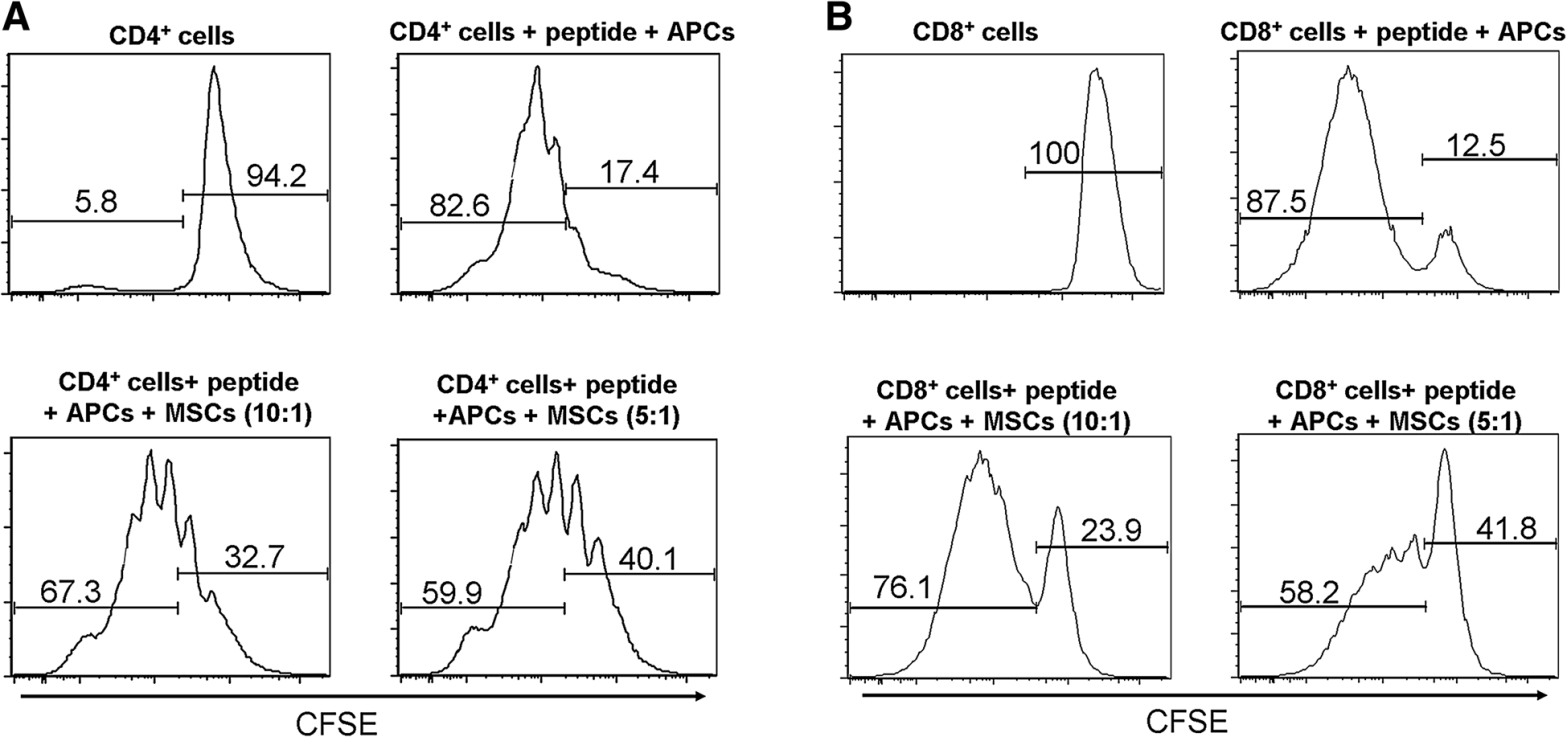
**hMSCs inhibit antigen-induced murine T-cell proliferation.** A total of 2 × 10^6^ purified antigen specific murine CD4^+^**(A)** and CD8^+^ T cells **(B)** were activated by their cognate peptides in the presence of antigen-presenting cells (APCs), and then co-cultured with human bone marrow-derived mesenchymal stem cells (hMSCs) in two different ratios (10:1 and 5:1). T cells were CFSE labeled on Day 0 and then harvested 72 hours later and analyzed by FACS. TCR transgenic CD4^+^ T cells (BDC2.5 T cells) were first gated using CD4 and TCR Vβ4 double staining, while CD8^+^ TCR transgenic CD8 T cells (8.3 T cells) were gated using CD8 and TCR Vβ8.1/8.2 double staining. The gates used to determine the percentage of undivided T cells (right hand bars) and divided T cells (left hand bars) are shown in each panel. The graphs are representative of four different individual experiments with similar results.

Murine TCR transgenic CD4^+^ and CD8^+^ T cells were isolated from NOD/BDC 2.5 mice and NOD/8.3 mice, respectively. They were co-cultured for three days together with irradiated (4,000 rads) total spleen cells from NOD/ShiLtj mice acting as APCs. The human MSCs were added to the culture and the proliferation of the T cells was monitored by CFSE dilution assay (Figure 1). After three days in culture, the control T cells that were not stimulated by their cognate peptides in the presence of APCs showed minimal or no proliferation, whereas the T cells that were stimulated by specific peptide showed substantial proliferation as indicated by CFSE staining. The optimal concentration of the peptide to stimulate the T cells was found to be 1 μg/ml, after multiple experiments in which different concentrations were used. T cells were co-incubated with human MSCs at different ratios. The T cells that we co-incubated with human MSCs at a ratio of 10:1 showed a decrease in proliferation (67.3% for CD4^+^ T cells and 76.1% for CD8^+^ T cells), as compared with T cells that we stimulated in the absence of MSCs (82.6% for CD4^+^ T cells and 87.5% for CD8^+^ T cells). Additionally, incubating the T cells with a higher ratio of MSCs (5:1) showed an even larger decrease in the percentage of divided cells (59.9% for CD4^+^ T cells and 58.2% for CD8^+^ T cells). We performed additional experiments using several other ratios (20:1, 50:1, 100:1) and did not see any immunosuppression using these lower ratios (data not shown). These results show an inhibitory effect of hMSCs on murine T cells in an *in vitro* setting. The extent of inhibition is dependent on the ratio of hMSCs to T cells, with more MSCs per T cell causing more inhibition.

**Figure 2:**
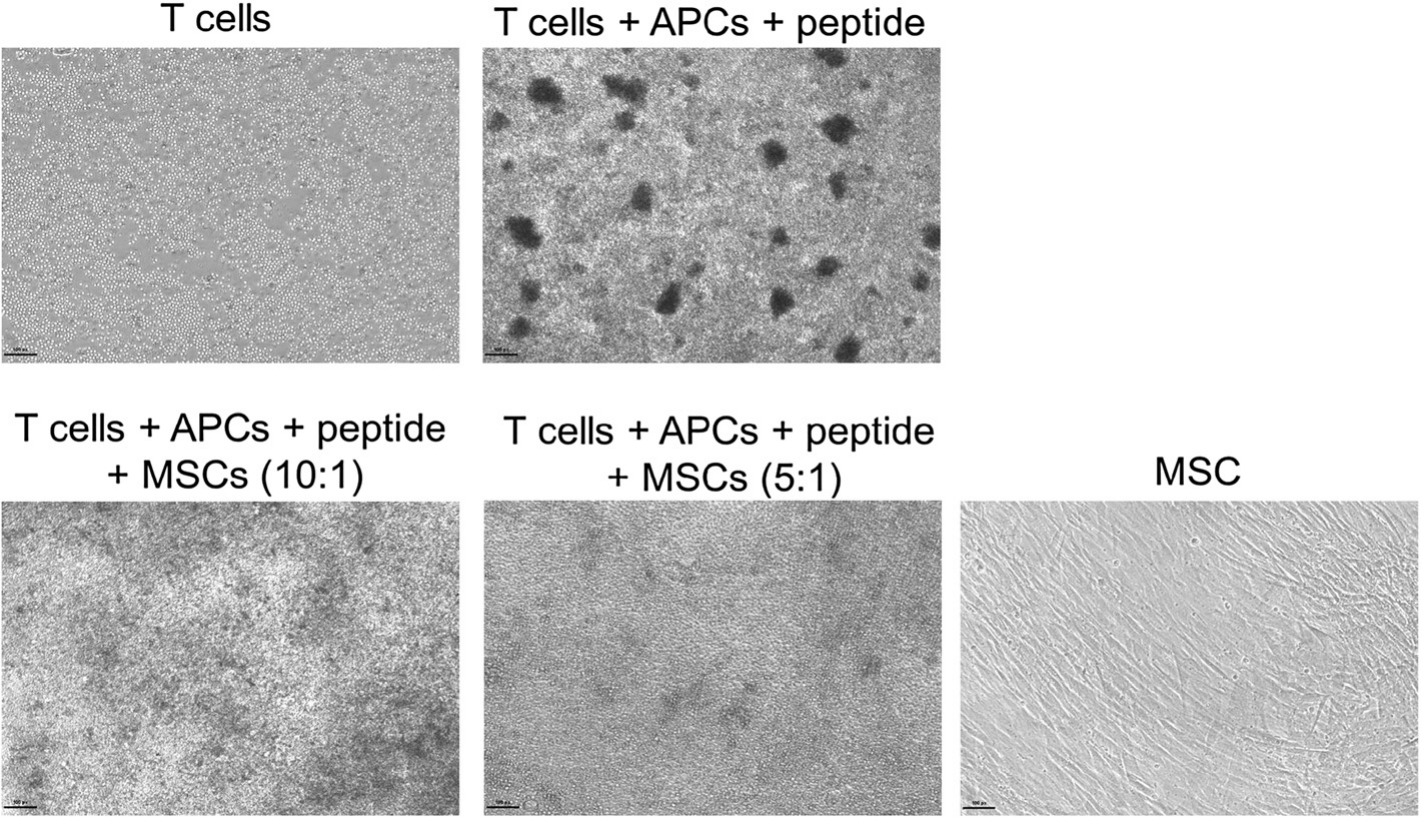
**hMSCs inhibit formation of clusters following T cell activation.** The figure shows bright field images (40X) of CD8^+^ T cells un-stimulated or stimulated with specific peptide (upper panels), or incubated with human bone marrow-derived mesenchymal stem cells (hMSCs) in two ratios (lower panels) for 72 hours. Untreated hMSCs are shown on the lower right. The results are representative of more than 10 different individual experiments with similar findings. Scale bars represent 100 μm.

After three days in culture, the cells were examined by microscope. The stimulated CD8^+^ T cells show a change in morphology (formation of large clusters) as opposed to the non-stimulated T cells (Figure 2). Co-incubation with hMSCs shows a dramatic change in appearance, the clusters completely disappear, no clusters can be found at both 10:1 and 5:1 ratio.

### hMSCs affect the expression of different murine T cell activation markers

**Figure 3:**
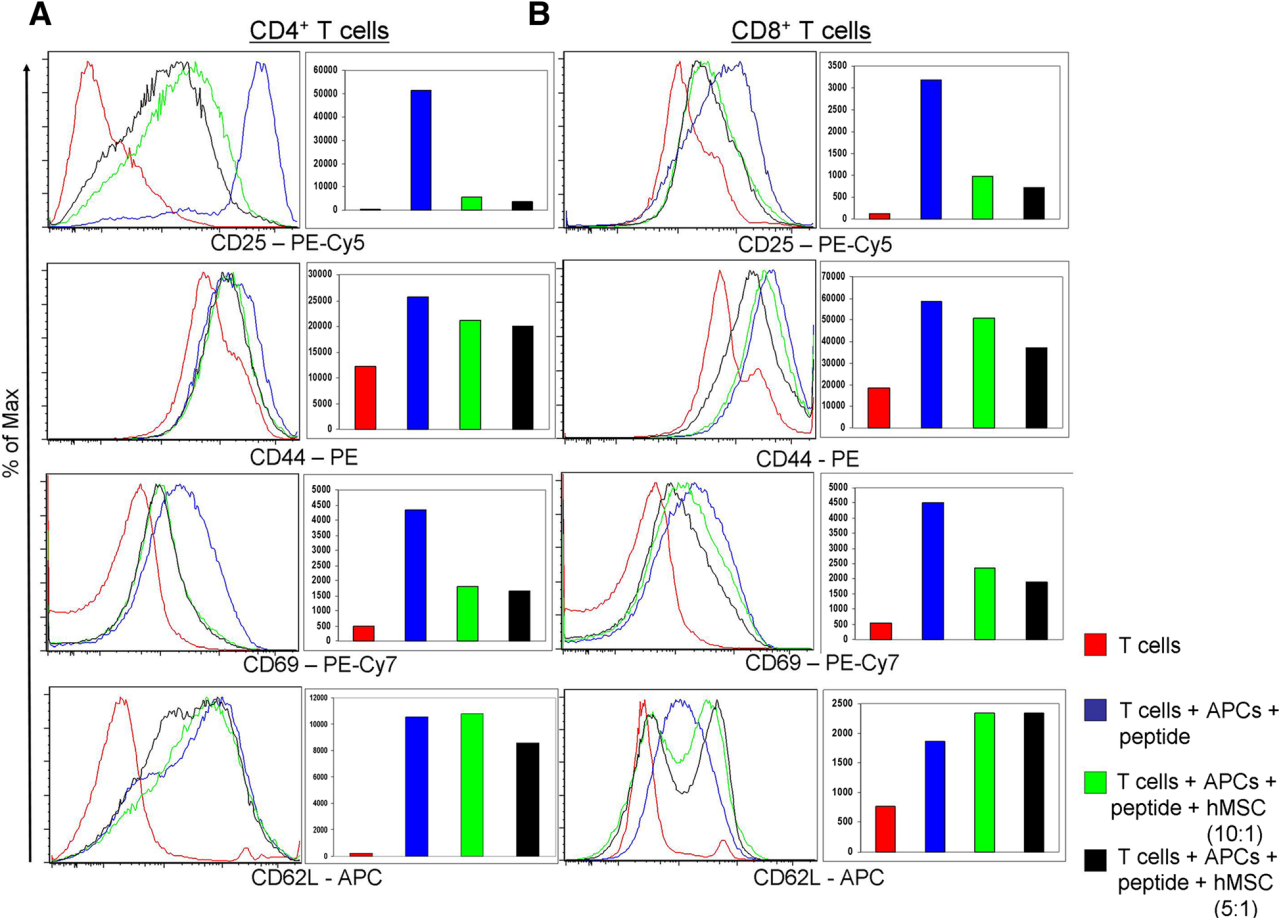
**hMSCs effect on different activation markers on murine antigen-specific T cells.** Murine CD4 **(A)** and CD8 **(B)** T cells were isolated and purified from lymph nodes and spleens of TCR transgenic mice using Miltenyi mouse CD4^+^ T cell/CD8^+^ T cell isolation kit and cultured for three days in the presence of specific peptides and APCs. Human bone marrow-derived mesenchymal stem cells (hMSCs) were added to the wells at the beginning of the culture in two ratios (1:10 and 1:5 hMSC to T cells). The cells were harvested and stained with antibodies specific for activation markers as indicated below each panel. Each graph shows overlays of histograms representing each condition as shown in the key: unstimulated T cells - red; stimulated T cells - blue; stimulated T cells + hMSC (ratio 10:1) - green; stimulated T cells + hMSC (ratio 5:1) - orange. The bar graphs depict the mean fluorescence intensity (MFI) of the analyzed surface markers on T cells in different treatment groups. All graphs are representative of three individual experiments with similar results.

Murine T cells were activated as described in the Materials and methods section and co-cultured with hMSCs. After three days in culture, cells were stained for various activation markers (Figure 3). In both CD4 and CD8 positive T cells, the analyzed markers show an increased expression after incubation with peptide and APC. CD25 expression was significantly decreased (greater than nine-fold decrease in mean fluorescence intensity (MFI)) after co-incubation with hMSCs in both ratios (10:1 and 5:1). CD69 showed a similar pattern of expression to CD25, being significantly inhibited (three-fold decrease in MFI) by hMSCs, whereas CD44 was only slightly decreased by hMSCs in both CD4^+^ T and CD8^+^ T cells. The effect on both CD25 and CD69 was hMSC dose-dependent, as greater inhibition of both markers was seen at a ratio of 5:1 compared to 10:1.

For CD62L we observed a differential effect of hMSCs on CD4^+^ T cells vs. CD8^+^ T cells. In both CD4^+^ T cells and CD8^+^ T cells with no hMSCs present, the expression of CD62L was up-regulated upon stimulation with cognate peptides. Interestingly, in CD4^+^ T cells the expression of CD62L is more homogeneous, whereas in CD8^+^ T cells we observed two peaks in the expression pattern, corresponding to a low-expressing population and a high-expressing population. The hMSCs down-regulated the expression of CD62L in CD4^+^ T cells (especially at the 5:1 ratio), as indicated by MFI; but in the CD8^+^ T cells, hMSCs co-incubation lead to an increase in the “high-expression” peak and a slight increase of MFI.

### hMSCs can affect the activation of murine T cells stimulated with anti-CD3/anti-CD28 beads

**Figure 4:**
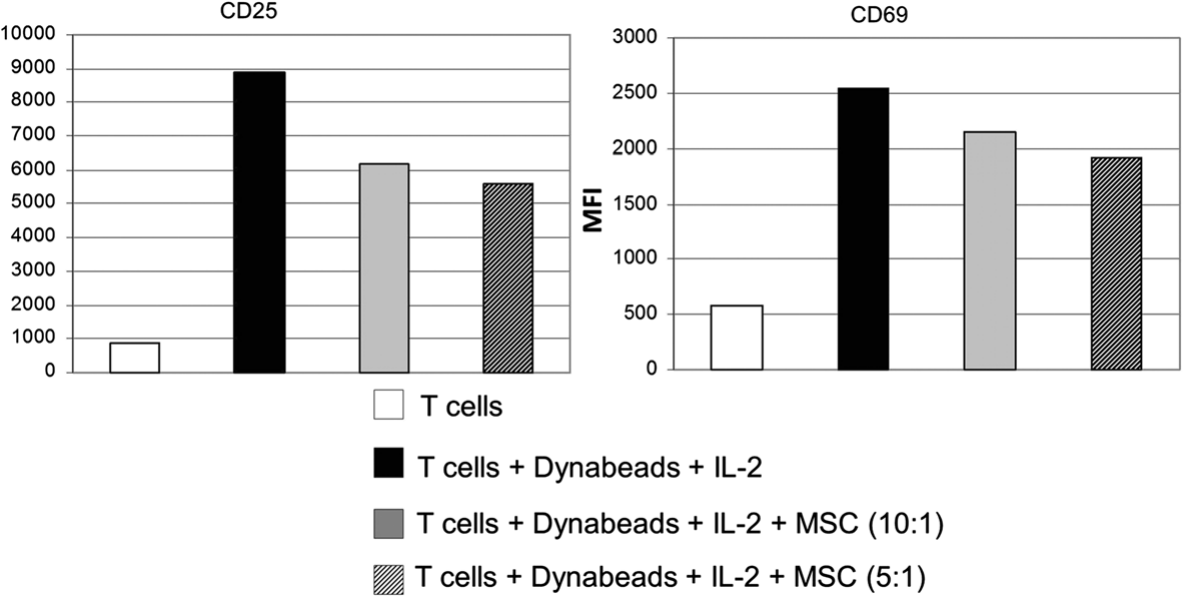
**hMSCs also inhibit the activation of T cells stimulated with anti-CD3/anti-CD28 beads.** Mouse Ag specific CD8^+^ T cells (8.3 T cells) were activated using anti-CD3/anti-CD28 Dynabeads (Invitrogen) (1:1 ratio) and IL-2 (2,000 U/ml) for three days in the presence or absence of human bone marrow-derived mesenchymal stem cells (hMSCs) at the ratio of 5:1 T cells:MSC. Expression of activation markers (CD25, CD69) was evaluated by flow cytometry and the mean fluorescence intensity (MFI) was shown for each marker. The values are representatives of three separate experiments.

In the literature, other methods have been used to evaluate the immunoregulatory property of hMSCs, some of them use the antigen-unspecific system, such as the anti-CD3/anti-CD28 induced T cell activation. To assess whether anti-CD3/anti-CD28 stimulated murine T cell activation can also be inhibited by hMSCs, we activated purified murine antigen-specific CD8 TCR transgenic T cells (8.3 T cells) with Dynabeads (Invitrogen) conjugated with anti-CD3/anti-CD28 mAbs at 1:1 ratio and analyzed the T cell activation markers (CD25, CD69) on the murine 8.3 T cells. As shown in Figure 4, hMSCs can also efficiently inhibit the anti-CD3/anti-CD28 induced up-regulation of CD25 and CD69 on murine antigen-specific CD8^+^ T cells.

Also, we have checked the impact of hMSCs on surface markers of already activated murine CD8^+^ T cells (8.3 T cells). As shown in Additional file 1: Figure S1, the hMSCs do not have an inhibitory effect on surface markers of activated T cells.

### CD8^+^ T cell apoptosis is affected by hMSCs

**Figure 5:**
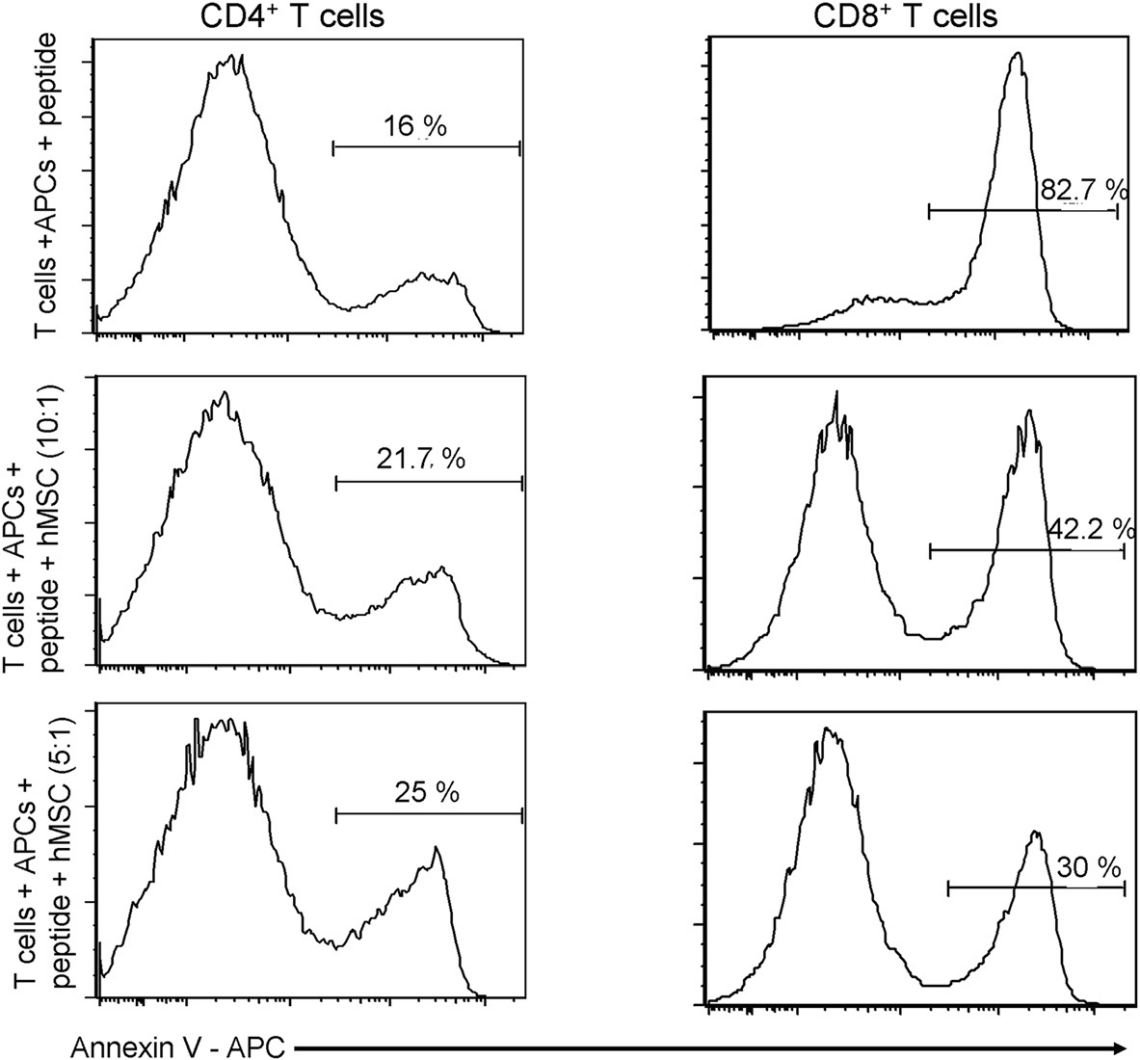
**hMSCs protect CD8**^**+ **^**T cells from apoptosis.** Negatively selected CD4 and CD8 murine T cells were co-cultured with hMSC in the presence of specific peptide and APCs for three days. Cells were stained with annexin V - APC and analyzed immediately on a FACS Canto II BD instrument. Results were analyzed using (Tree Star, Inc., Ashland, OR) software. The experimental conditions are shown to the left of each set of panels. The histograms are representative of three independent experiments with similar results. The horizontal bar shows the gate used to determine the percentage of apoptotic cells after treatment. hMSCs, human bone marrow-derived mesenchymal stem cells.

There are contradictory data in the literature concerning the effect of hMSCs on the viability of T cells. Some reports show that the hMSCs induce apoptosis when co-cultured together with human T cells, while others observe that hMSCs lead to a cell cycle arrest of the human T cells [13,24,25], but do not induce apoptosis. We wanted to determine whether hMSCs induce murine T cell apoptosis in our system; therefore, three days after co-culturing the T cells together with their cognate peptide and APC plus hMSCs, we performed an apoptosis assay. The cells were counted and then stained with annexin V and 7AAD. 7AAD negative and annexin V positive cells were defined as apoptotic. The results depicted in Figure 5 show annexin V expression in both populations analyzed (CD4^+^ and CD8^+^ T cells). Again, the results showed a differential effect of hMSC on murine CD4^+^ and CD8^+^ T cells.

Only a fraction of the CD4^+^ T cell population stained positive for Annexin V after being stimulated with a specific peptide and APCs (16%). Co-culture with hMSCs at both 10:1 and 5:1 ratios slightly increased the number of annexin V positive cells (21.7% to 25%), supporting the idea that hMSCs only marginally affect the viability of CD4^+^ T cells.

Conversely, stimulated CD8^+^ T cells showed a significant number of apoptotic cells (82.7%) after three days in culture. Incubation with hMSCs led to a decreased number of apoptotic cells (42.2%, 30%), suggesting that hMSCs seem to contribute to a better survival of these CD8^+^ T cells in culture. The effect seems to be dose dependent, with the ratio of 5:1 showing a more significant effect on the T cells.

### hMSCs decrease the level of mRNA expression of two important transcription factors

**Figure 6:**
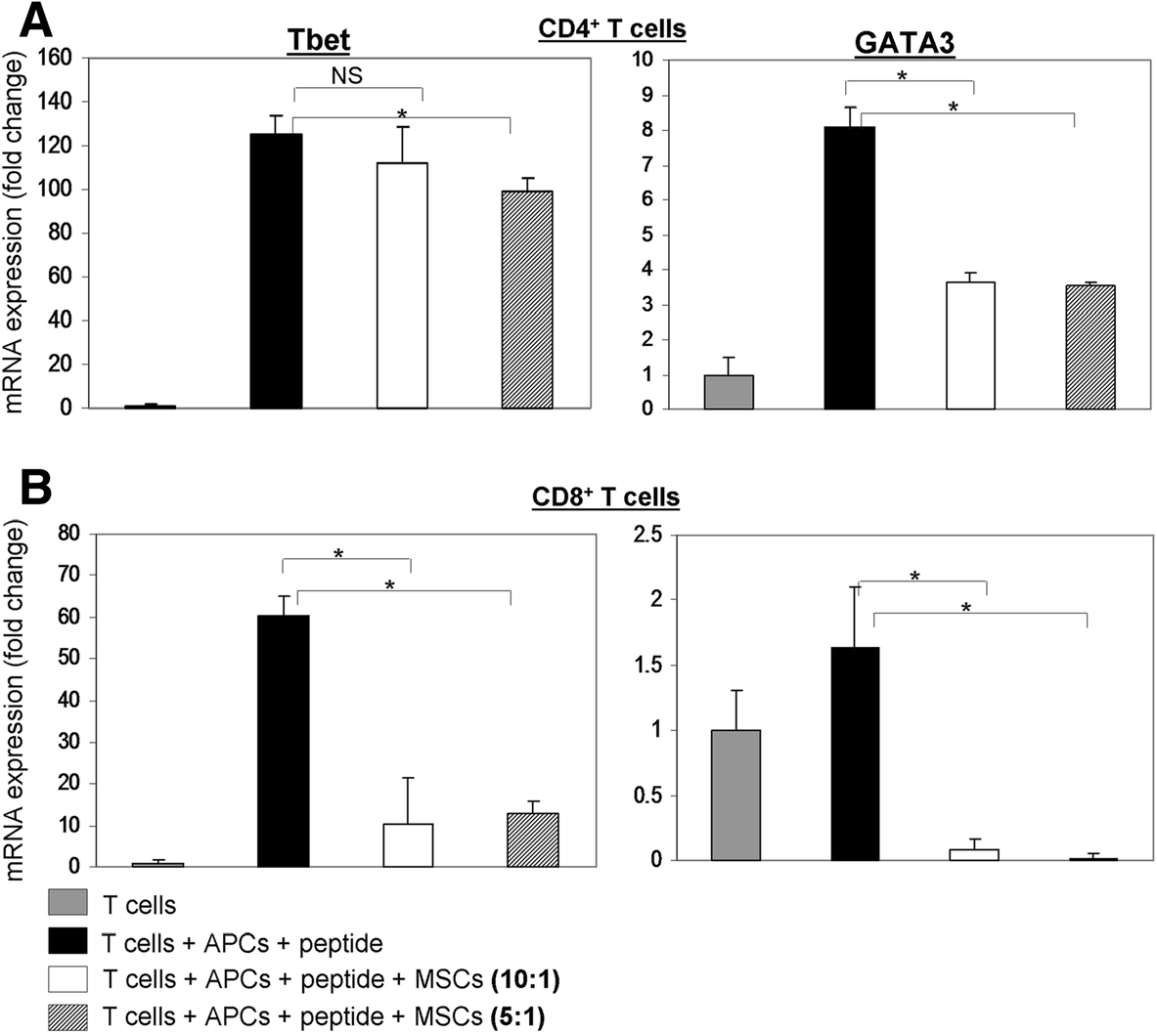
**hMSCs inhibit expression of T-bet and GATA-3 in murine CD4**^**+ **^**and CD8**^**+ **^**T cells.** Total RNA was extracted from murine T cells 48 hrs after stimulation with specific peptides and co-culturing with human bone marrow-derived mesenchymal stem cells (hMSCs). The RNA was reverse transcribed into cDNA and specifically amplified using probes for murine T-bet and GATA-3. Bars in each panel represent fold change of mRNA expression as compared with un-stimulated T cells. Results for CD4^+^ T cells are shown in panel **A** and for CD8^+^ T cells in panel **B**. Every assay was done in triplicate, values are expressed as the mean and SD of triplicates. Statistical analysis was carried out by applying the Student’s *t-* test. *indicates *P* <0.05 for differences between conditions shown by the horizontal brackets. The results shown represent one out of three independent experiments with similar results.

In order to further examine the effects of hMSCs on murine T cells at the molecular level, we then analyzed mRNA expression patterns. Total RNA was extracted 48 hrs after co-culturing the T cells with hMSC, and then subjected to reverse transcription and real time RT-PCR with specific probes for murine T-bet and GATA-3 transcription factors. There is a significant increase in expression of both transcription factors after activation of CD4^+^ T cells, while after activation of CD8^+^ T cells we only observed notably increased expression of T-bet (Figure 6). T-bet gene expression level was increased 125 times in CD4^+^ T cells and approximately 60 times in CD8^+^ T cells compared with the un-stimulated cells. GATA-3 gene expression level was increased 8 times in CD4^+^ T cells and 1.7 times in CD8^+^ T cells compared with the un-stimulated T cells. Stimulation in the presence of hMSCs led to decreased transcription factor expression in both CD4^+^ and CD8^+^ T cell population, with GATA-3 showing a more significant reduction in gene expression than T-bet in CD4^+^ T cells. It has been previously reported that hMSCs have a preferential inhibitory activity on Th1 cells [26], but in our system GATA-3 (a hallmark transcription factor for the Th2 subset) was more significantly affected by hMSCs, while T-bet was affected to a lesser extent. The inhibitory effect of hMSCs on both transcription factors is in agreement with the effect on activation marker expression at the surface and to our knowledge is the first report of the effect of hMSCs on murine T cells at the molecular level. The results were consistent, as we repeated the experiments three times and found similar results.

### hMSCs affect the secretion of cytokines by CD4^+^ and CD8^+^ T cells

**Figure 7:**
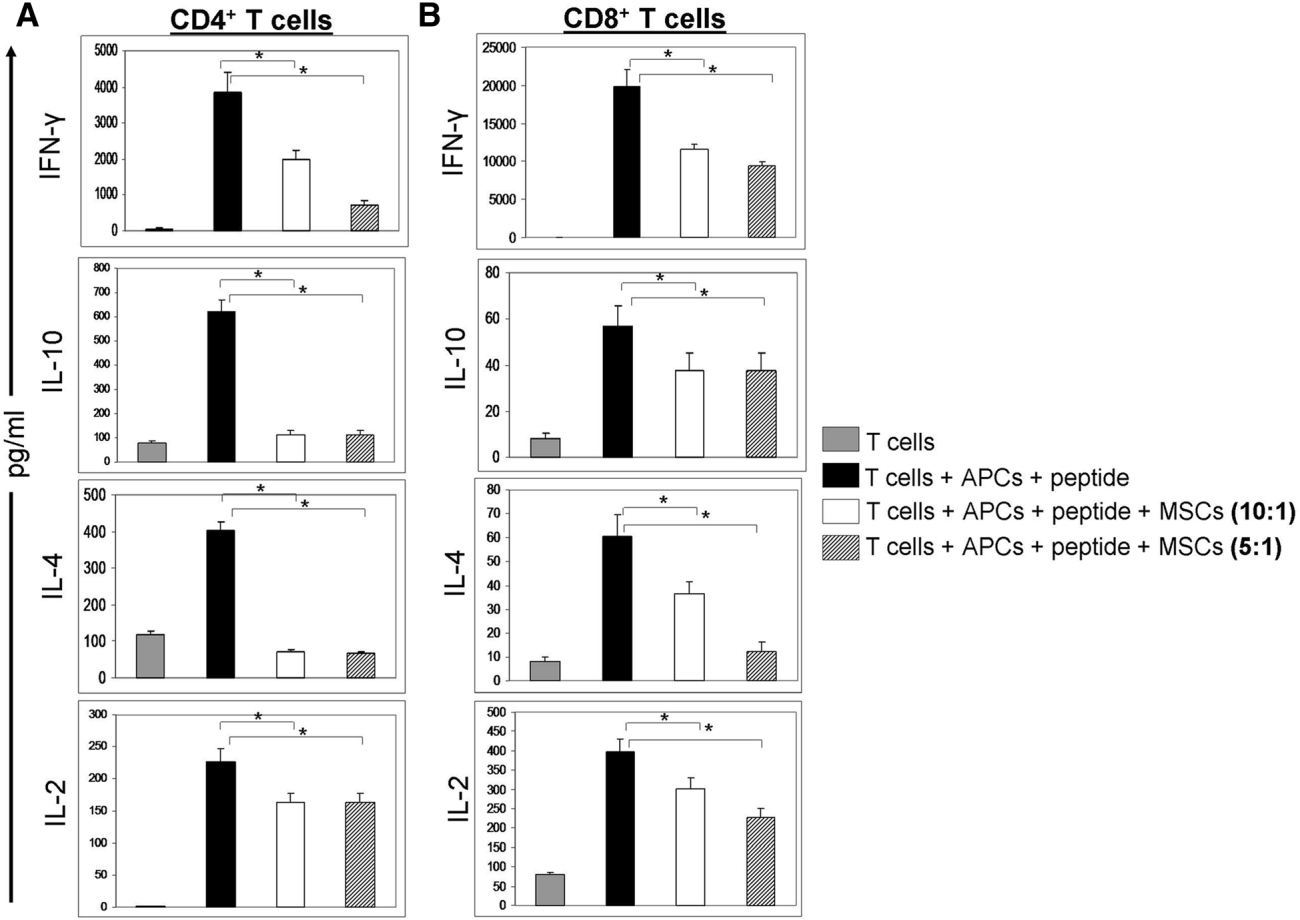
**hMSCs inhibit secretion of Th1/Th2 cytokines by antigen stimulated murine T cells.** Murine CD4^+^ (panel **A**) or CD8^+^ (panel **B**) T cells were cultured in the same conditions as described earlier (2 × 10^6^ cells/well), together with their specific peptide and APCs. Human bone marrow-derived mesenchymal stem cells (hMCSs) were added to the T cells in the ratios 1:10 and 1:5 and supernatants were collected 72 hours later. Cytokine secretion was quantified and results are shown as pg/ml on the y axis. Shown are representative data out of two independent experiments with similar results. The samples were set up in triplicates in each experiment. Cytokine concentrations are represented in pg/ml for each sample, and results are expressed as the mean and SD of triplicates. *indicates *P* <0.05 for differences between conditions shown by the horizontal brackets.

It has been shown that hMSCs inhibit the production of various cytokines by T cells both *in vitro* and *in vivo*[27]. We wanted to assess if cytokine production is also inhibited in our system. Supernatants were collected from samples 72 hours after the initial setup. We analyzed six murine cytokines using the Th1/Th2 multiplex ELISA. The levels of all six murine cytokines analyzed: IL-10, IFN-γ, IL-12, IL-5, IL-4 and IL-2 showed similar patterns of expression (the values of IL-12 and IL-5 were very low; therefore, the data were now shown). As expected, the cytokine concentrations were higher in the activated T cells as compared with the non-stimulated cells. After co-culturing with hMSCs the amount of murine cytokines secreted in the medium was significantly lower than seen for activated T cells. This holds true for both the CD4^+^ and CD8^+^ populations. IFN-γ was the most abundantly secreted cytokine after stimulation of both CD4^+^ and CD8^+^ T cells (Figure 7). The concentration of IFN-γ secreted by the activated CD8^+^ T cells (19,000 pg/ml) was significantly higher than that secreted by the CD4^+^ T cells (3,500 pg/ml). The hMSCs lowered the concentration of secreted IFN-γ by both CD4^+^ and CD8^+^ T cells in a dose dependent manner. This is in agreement with previous reports showing a reduction in the cytokine secretion ability of T cells after they are incubated with hMSCs [11]. Differing from previous reports showing that hMSCs promote production of Th2 cytokines [24,26], we found that in this system the clonal murine CD4^+^ and CD8^+^ T cells that were incubated with hMSCs secreted a lower amount of IL-10 (a Th2 cytokine) when compared with the activated T cells. The effect was more evident on CD4^+^ T cells, as they are better IL-10 secretors than CD8^+^ T cells. All these results clearly demonstrate the inhibitory effect that hMSCs have on murine T cells in an *in vitro* system. Since hMSCs inhibited IFN-γ secretion by both CD4 and CD8 T cells in a dose dependent manner, this assay could be used to quantitatively measure the immunosuppressive activity of hMSCs.

Since Treg and Th17 cells are also important regulators in the immune system, the effects of hMSCs on these T cell subsets were checked as well. As shown in the Additional file 2: Figure S2, we did not see remarkable changes in the frequencies of Treg and Th17 cells in the presence of hMSCs.

### Human fibrosarcoma cells do not have any effect on the proliferation and activation of murine T cells

To demonstrate that the inhibitory effect that hMSCs have on murine T cells is specific to the hMSCs, we also used a different adherent human cell line in our experiments, HT-1080 (human fibrosarcoma cell line). These cells have similar morphological features to hMSCs, without having any known MSC-like properties (the multi-potent ability to differentiate into osteocytes, chondrocytes and adipocytes); therefore, they are used as controls in our experiments. We used these cell lines in the same ratios as the hMSCs, and the same experimental settings.

**Figure 8:**
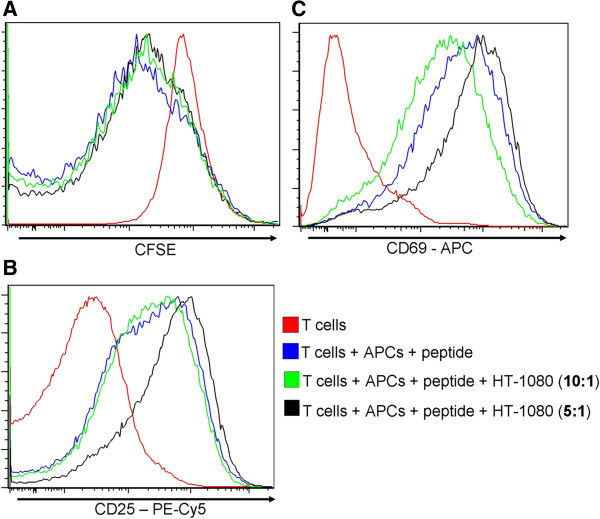
**Human fibrosarcoma cells do not affect mouse T cells proliferation and activation.** Murine CD4^+^ T cells were labeled with CFSE and incubated with their cognate peptide and APCs for three days with or without human fibrosarcoma cells HT-1080. A total of 72 hours later, the cells were analyzed for the CFSE profile **(A)** and for the expression of two activation markers: CD25 **(B)** and CD69 **(C)**. The unstimulated CD4^+^ T cells are represented by a red histogram, the stimulated T cells by a blue histogram and the cells that were co-cultured with human epithelial cells are represented as green (ratio 10:1) and orange (ratio 5:1) histograms. The results are representative of three independent experiments with similar results.

The results shown in Figure 8 are representative of three different experiments conducted with each cell line. First, proliferation as shown by CFSE staining is not affected by the control cell line, with the percentage of undivided cells remaining the same even after incubation with human fibrosarcoma cells in a ratio of 5:1. Second, unlike hMSCs, both CD25 and CD69 expression on CD4^+^ T cells were not inhibited by the control cell line. On the contrary, there seems to be a slight increase in the expression of these two activation markers in CD4^+^ T cells when HT-1080 is present at one per 5 T cells, but not one HT-1080 per 10 T cells. The same effect was observed in CD8^+^ T cells (data not shown). It has been suggested in the literature that human fibroblasts have an inhibitory effect on the immune system, similar to hMSCs [28], albeit by a different mechanism than mesenchymal stem cells. We tested that hypothesis by using primary human dermal fibroblasts in our experiments. The fibroblasts do not have any inhibitory effect on the T cells, supporting our claim that the immuno-suppressive effect in our system is specific to the hMSCs (data not shown).

### The immuno-suppressive effect of hMSCs is cell contact-dependent

There has been widespread interest in elucidating the mechanism by which hMSC act as suppressors of the immune system. It is accepted that hMSCs act by cell-cell contact inhibition and by secreting soluble factors *in vitro* as well as *in vivo*[29].

We aimed at understanding the mechanism by which hMSCs affect the proliferation and activation of murine antigen specific T cells in our *in vitro* system. The CD4^+^ T cells or CD8^+^ T cells were cultured together with their cognate peptides and APCs in the bottom wells of a 24-well transwell system plate. In the upper wells we cultured the hMSCs in the same ratios as previously.

**Figure 9:**
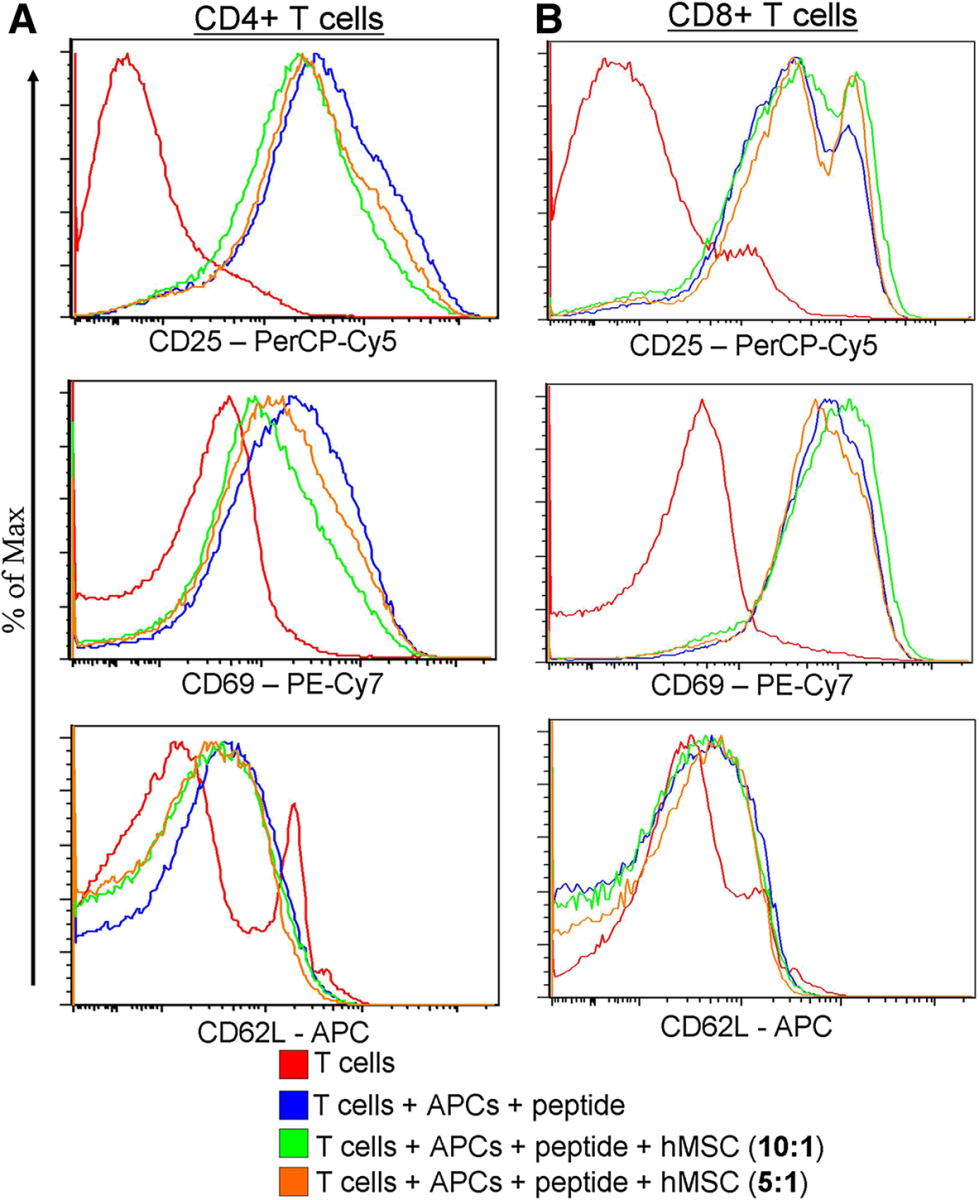
**Effects of hMSCs on murine T cells are cell-contact dependent.** A total of 2 × 10^6^ CD4^+^ (panel **A**) or CD8^+^ (panel **B**) T cells were cultured on the bottom well of a transwell system together with the specific peptide and APCs. The human bone marrow-derived mesenchymal stem cells (hMSCs) were cultured in the upper well of the transwell system and the medium was shared between the two compartments. A total of 72 hours later the T cells were stained with antibodies against CD25, CD69 and CD62L and analyzed using a FACS Canto flow cytometer. Unstimulated T cells are represented by a red histogram, stimulated T cells by a blue histogram and T cells that were co-cultured with hMSCs are represented as green (ratio 10:1) and orange (ratio 5:1) histograms. The results are representative of three independent experiments, all with similar results.

After 72 hours, we analyzed activation marker expression on the surface of T cells (Figure 9). All markers analyzed were clearly not affected by the hMSCs present in the upper compartment of the transwell system. This is in disagreement with published reports indicating that soluble factors are responsible for the immuno-suppressive effect of hMSCs [30], as we show no difference between the activated T cells and the T cells that were cultured in a transwell system together with hMSCs. This may be due to the fact that the experiments we performed were showing a cross-species effect of hMSCs, while other reports based their findings on a syngeneic or allogeneic reaction.

## Discussion

Due to their immunosuppressive activities, hMSCs have been used in many investigational clinical trials to investigate their potential to treat immunological disorders or inflammation-mediated pathological lesions, including Crohn’s disease, T1D and GVHD (reviewed in [16]). They have also been investigated in co-transfer experiments intended to improve the engraftment of allogeneic pancreatic islet transplant [31] and hematopoietic stem cells [32,33]. Because of the heterogeneous nature of hMSCs, the establishment of quantitative bioassays that could detect differences between hMSCs from different donors and passages would potentially be of great value for manufacturing and MSC product assessment purposes. Currently, there is an increasing need to develop more sensitive, accurately quantitative cell-based or *in vitro* bioassays suitable for detecting small range differences in immune-inhibitory activity of hMSCs from different donors or at different passages in tissue culture, or under different tissue culture expansion conditions. For example, the traditionally used mixed lymphocyte reaction (MLR) is a semi-quantitative, or relatively qualitative, rather than quantitative assay; the result may be affected by many factors such as the mismatch extent of donor and recipient MHC, gender and age of donor, as well as the previous and current infectious disease status. With such inherent variability, it can be very challenging to capture minor differences in immunosuppressive activity between different lots of hMSC products using the traditional MLR method.

It has been established that the immune inhibitory activity of MSCs works across allogeneic barriers, and it has also been reported that human MSCs can home to tissues, survive and function to various extents in xenogenic models, such as in mice and rats [18,26,34-36]. Therefore, it is likely the immune inhibitory activity of the MSCs will work across species, at least partially. For this reason, we explored development of quantitative immune inhibition assays using clonal murine T cell populations (derived from TCR transgenic mice), known peptide antigens, and MSCs from different human donors. Compared with other existing systems, the advantages of this system include genetic and age variation between human T cell donors is eliminated; the murine donors are kept under specified pathogen free (SPF) conditions; the mouse TCRs are monoclonal with known antigen specificity; and these clonal mouse T cells are reliably available in essentially unlimited supply.

Through the work presented in the present study, we discovered that hMSCs can inhibit the activation and effector functions of mouse Ag specific T cells in response to stimulation with cognate peptide antigens as well as anti-CD3/anti-CD28. Many aspects of T cell activation are affected, such as cell surface markers CD25, CD44, CD62L, CD69, proliferation and cytokine production. The effects are intrinsic to hMSCs, since control cell lines (fibrosarcoma, hepatocellular carcinoma, fibroblasts) do not exert these activities. To our knowledge, this is the first report to demonstrate the cross-species effect of hMSCs on clonal murine T cells when they are stimulated with cognate peptide antigens.

Such an *in vitro* bioassay may be useful to assess the immunosuppressive activity in human MSCs from different donors, or to assess the effect of different tissue culture expansion conditions of the MSCs from the same donor on their immuosuppressive activity. It might be particularly valuable to researchers who have access to make use of the animal resources as a supplementary method when inter-donor (patient) variance is a major interference issue. Taking into consideration the fact that obtaining TCR transgenic animals and purifying mouse T cells is a relative cumbersome and probably not the most cost-effective method, this method will not be applicable to a routine cell therapy laboratory. However, if acceptable reproducibility of the assay can be achieved through optimization, potentially it might assist in informative comparison of MSC lots and different manipulation conditions.

Despite the fact that these results parallel previous findings with allogeneic MSCs, some of the results obtained in this study are not completely consistent with earlier reports. For example, it has been reported that MSCs preferentially skew the immune response toward Th2 over Th1 by inhibiting the production of TNF-α and IFN-γ by CD4^+^ T cells (helper T cells) and CD8^+^ cytotoxic T cells, while they up-regulate the expression of IL-10 and IL-4 by CD4^+^ and CD8^+^ T cells [24]. From these results it would be expected that hMSCs inhibit the production of IFN-γ and the expression of transcription factor T-bet. However, we also observed inhibition of IL-10 and the Th2 transcription factor GATA-3. This could be due to differences between our model systems (that is, the cross-species use of hMSCs with murine T cells in this study, versus allogeneic hMSCs with human T cells; clonal T cells versus polyclonal T cells, and so on). Also, it is known that when TCR signal transduction pathways are triggered with rigorous stimuli (such as PMA and ionomycin, anti-CD3 Ab) in T lymphocytes, both Th1 and Th2 cytokines are released [37,38], and GATA-3 expression can be turned on by TCR signals [39,40]. Thus, it is most likely that the inhibition of Th2 cytokines as well as GATA-3 expression that we observed is merely a reflection of hMSC-mediated inhibition of TCR signaling and T cell activation.

Although the mechanisms of immune regulation have been extensively studied they are still not fully elucidated. It is well-known that allogeneic MSCs can inhibit the activation of T cells following stimulation with mitogenic or allogeneic stimulation (PHA, Con A, CD3, allogeneic PBL and so on) [9,10,41]. Also, peptide antigen-stimulated T cell activation can be inhibited [11]. Several studies have suggested that these immunoregulatory effects require an initial cell-cell contact phase, but that suppressive signaling is mediated by soluble factors including transforming growth factor beta 1 (TGF-β1) [10], indoleamine 2,3-dioxygenase (IDO) [42], prostaglandin E2 (PGE2) [43], nitric oxide (NO) [44], heme oxygenase-1 (HO-1) [45], and insulin-like growth factor-binding proteins [46]. In our study, the immunosuppression by hMSCs upon both CD4^+^ and CD8^+^ T cells seems to be predominantly mediated by a cell-cell contact mechanism. Perhaps the cross-species effects of hMSCs are biologically different from those in syngeneic or allogeneic systems studied by others, which need to be further clarified. We plan to test the immunosuppressive activities of mouse MSCs on murine CD4^+^ and CD8^+^ T cell clones, to investigate whether the effects we saw in this study are simply due to the difference of cytokines or soluble factors between mouse and human species.

There is controversy as to whether MSCs inhibit T cell proliferation by inducing apoptosis or not. In our experiments, we noticed a slight increase in Annexin V expression when CD4^+^ T cells were incubated with hMSCs, in agreement with one report that hMSCs can induce apoptosis via a mechanism involving IDO and IFN-γ [47]. Other studies have reported that hMSCs inhibit the proliferation of T cells by non-apoptotic mechanisms, such as cell cycle arrest [13,48]. Based on previous reports, it is surprising to see in our system that hMSCs can decrease apoptosis in activated murine CD8^+^ T cells. We hypothesize that hMSCs might provide certain soluble growth factors or cell-cell contact signals that favor the survival of CD8^+^ T cells or prevent them from activation-induced cell death. Further studies are needed to gain better understanding of the underlying mechanism.

**Figure 10:**
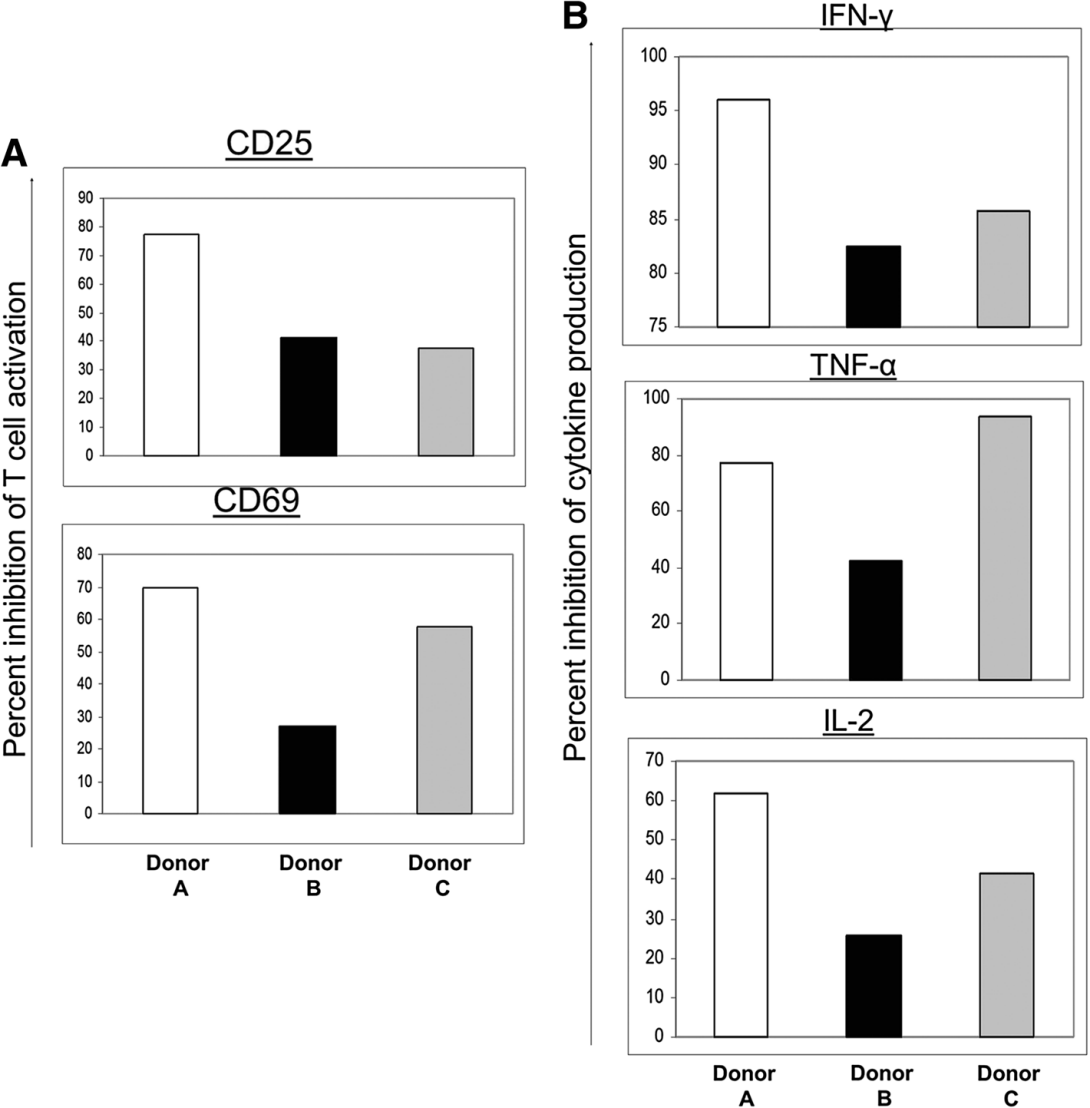
**hMSCs from three different donors effect T cell activation and cytokine production.** Murine CD8^+^ T cells (8.3 TCR transgenic T cells) were isolated and purified from lymph nodes and spleens of NOD 8.3 TCR transgenic mice using Miltenyi mouse CD8^+^ T cell isolation kit and cultured for three days in the presence of specific peptides and antigen-presenting cells (APCs). Human bone marrow-derived mesenchymal stem cells (hMSCs) from three different donors (donor A, B and C) were added to the wells at the beginning of the culture at a ratio of 5:1 (T cells:hMSC). The cells were harvested, activation markers (CD25, CD69) were evaluated by flow cytometry and the percent inhibition of activation-induced mean fluorescence intensity (MFI) increase was calculated as follows: (MFI increase of activated T cells without MSCs - MFI increase of T cells with MSCs)/MFI increase of activated T cells without MSCs × 100%. Percent of inhibition was shown for all three donors (panel **A**). Cytokine concentrations in the culture supernatant were analyzed using a multiplex ELISA assay and the percent inhibition in cytokine production was shown for all three donors (panel **B**). The graph is representative of two independent experiments with similar results.

In all the experiments presented in this study we used hMSCs isolated from a single donor (PCBM 1632), expanded to passage number 3. Experiments with human MSCs from several more donors demonstrated similar immunosuppressive activities against murine clonal T cells (our unpublished data). An example is shown in Figure 10. Further studies are currently underway to determine the molecular mechanism of immunosuppression, as well as to investigate whether further passaging the hMSCs has any effect on their immune suppressive function.

Since experimental results obtained from *in vitro* systems do not always reflect the *in vivo* environment, they need to be confirmed by *in vivo* findings. Ongoing experiments are being performed in our group to further evaluate the immuno-suppressive function of hMSCs in an *in vivo* murine model of autoimmune type 1 diabetes. Previously, MSCs obtained from healthy mice have been shown to delay the onset of diabetes in non-obese diabetic (NOD) mice [49]. Similarly, human MSCs lowered blood glucose levels in the STZ- (streptozotocin-) treated diabetic mice relative to untreated controls [18]. Once our *in vivo* studies are finished, we may be able to correlate the *in vivo* inhibitory functions of hMSCs with their *in vitro* activities, and even identify potential biomarkers which can be used to predict the *in vivo* efficacy before hMSC engraftment.

## Conclusions

In summary, we established a system where the immunosuppressive activity of hMSCs can be measured using murine clonal T cells; several biomarkers were identified which can be used to quantify the immunosuppressive activities of hMSCs, such as CD25, CD44, CD62L, CD69, proliferation, gene expression and cytokine production. Among these markers, cytokine measurement is most quantitative and easier to standardize, thus it could potentially contribute to an informative comparison of MSC lots and their potential manipulation.

## Abbreviations

APC: Antigen-presenting cell; GVHD: Graft versus host disease; hMSCs: Human bone marrow-derived mesenchymal stem cells; IFN: Interferon; IL: Interleukin; MFI: Mean fluorescence intensity; MHC: Major histocompatibility complex; MLR: Mixed lymphocyte reaction; MSCs: Mesenchymal stem cells; TCR: T cell receptor.

## Competing interests

The authors declare that they have no competing interests.

## Authors’ contributions

SRB, CN and CHW conceived and designed the experiments.. CN and JLS performed the experiments. CN, JLS, SRB and CHW analyzed the data. CN and CHW wrote the paper. All authors read and approved the final manuscript.

## Supplementary Material

Additional file 1: Figure S1 hMSCs do not affect surface markers on already activated T cells. Mouse CD8^+^ Ag specific T cells (8.3 T cells) were stimulated with anti-CD3/CD28 Dynabeads (Invitrogen) (1:1 ratio of T cells: beads) and human IL-2 (2,000 U/ml) for three days. Three days later, beads were removed, T cells were then re-stimulated with IGRP peptide-pulsed irradiated spleen cells, and human MSC were added to the culture at two different ratios: 10:1 and 5:1 and further incubated for an additional three days. Activation markers CD25 and CD69 for mouse 8.3 T cells were evaluated and the mean fluorescence intensity was plotted for each condition. The values are representative of three different experiments. Click here for file

Additional file 2: Figure S2 The presence of hMSCs does not lead to significant changes of Th17/Treg subsets. Purified mouse CD4^+^ Ag specific T cells (BDC2.5 T cells) were cultured in the presence of Antigen Presenting Cells and their cognate peptide for three days. hMSC were co-cultured with the mouse T cells at a ratio of 5:1 T cells: MSC. For measuring Th17 cells, T cells were stimulated with PMA (50 ng/ml) and Ionomycin (1 μg/ml) in the presence of Golgi-Plug at 37°C for five hours. Then surface staining with anti-CD4 and anti-TCR Vβ4 Abs, permeabilization/fixation (using BD Cytofix/Cytoperm kit) and Intracellular staining for mouse Foxp3 (eFluor450) and IL-17A (PE) was performed according to the manufacture’s instruction (BD). The histograms are representative of two separate experiments. Click here for file

## References

[B1] Pittenger MF Mackay AM Beck SC Jaiswal RK Douglas R Mosca JD Moorman MA Simonetti DW Craig S Marshak DR Multilineage potential of adult human mesenchymal stem cells Science 1999 284 143 147 10.1126/science.284.5411.143 10102814

[B2] Liu ZJ Zhuge Y Velazquez OC Trafficking and differentiation of mesenchymal stem cells J Cell Biochem 2009 106 984 991 10.1002/jcb.22091 19229871

[B3] Gonzalez MA Gonzalez-Rey E Rico L Buscher D Delgado M Treatment of experimental arthritis by inducing immune tolerance with human adipose-derived mesenchymal stem cells Arthritis Rheum 2009 60 1006 1019 10.1002/art.24405 19333946

[B4] Dominici M Le Blanc K Mueller I Slaper-Cortenbach I Marini F Krause D Deans R Keating A Prockop D Horwitz E Minimal criteria for defining multipotent mesenchymal stromal cells. The International Society for Cellular Therapy position statement Cytotherapy 2006 8 315 317 10.1080/14653240600855905 16923606

[B5] Delorme B Ringe J Pontikoglou C Gaillard J Langonne A Sensebe L Noel D Jorgensen C Haupl T Charbord P Specific lineage-priming of bone marrow mesenchymal stem cells provides the molecular framework for their plasticity Stem Cells 2009 27 1142 1151 10.1002/stem.34 19418444

[B6] Li TZ Kim JH Cho HH Lee HS Kim KS Lee SW Suh H Therapeutic potential of bone-marrow-derived mesenchymal stem cells differentiated with growth-factor-free coculture method in liver-injured rats Tissue Eng Part A 2010 16 2649 2659 10.1089/ten.tea.2009.0814 20367252

[B7] Ortiz LA Dutreil M Fattman C Pandey AC Torres G Go K Phinney DG Interleukin 1 receptor antagonist mediates the antiinflammatory and antifibrotic effect of mesenchymal stem cells during lung injury Proc Natl Acad Sci U S A 2007 104 11002 11007 10.1073/pnas.0704421104 17569781 PMC1891813

[B8] Horwitz EM Prockop DJ Fitzpatrick LA Koo WW Gordon PL Neel M Sussman M Orchard P Marx JC Pyeritz RE Brenner MK Transplantability and therapeutic effects of bone marrow-derived mesenchymal cells in children with osteogenesis imperfecta Nat Med 1999 5 309 313 10.1038/6529 10086387

[B9] Bartholomew A Sturgeon C Siatskas M Ferrer K McIntosh K Patil S Hardy W Devine S Ucker D Deans R Moseley A Hoffman R Mesenchymal stem cells suppress lymphocyte proliferation *in vitro* and prolong skin graft survival *in vivo* Exp Hematol 2002 30 42 48 10.1016/S0301-472X(01)00769-X 11823036

[B10] Di Nicola M Carlo-Stella C Magni M Milanesi M Longoni PD Matteucci P Grisanti S Gianni AM Human bone marrow stromal cells suppress T-lymphocyte proliferation induced by cellular or nonspecific mitogenic stimuli Blood 2002 99 3838 3843 10.1182/blood.V99.10.3838 11986244

[B11] Krampera M Glennie S Dyson J Scott D Laylor R Simpson E Dazzi F Bone marrow mesenchymal stem cells inhibit the response of naive and memory antigen-specific T cells to their cognate peptide Blood 2003 101 3722 3729 10.1182/blood-2002-07-2104 12506037

[B12] Le Blanc K Tammik C Rosendahl K Zetterberg E Ringden O HLA expression and immunologic properties of differentiated and undifferentiated mesenchymal stem cells Exp Hematol 2003 31 890 896 10.1016/S0301-472X(03)00110-3 14550804

[B13] Glennie S Soeiro I Dyson PJ Lam EW Dazzi F Bone marrow mesenchymal stem cells induce division arrest anergy of activated T cells Blood 2005 105 2821 2827 10.1182/blood-2004-09-3696 15591115

[B14] Prockop DJ Repair of tissues by adult stem/progenitor cells (MSCs): controversies, myths, and changing paradigms Mol Ther 2009 17 939 946 10.1038/mt.2009.62 19337235 PMC2835176

[B15] ClinicalTrials.gov: a service of the U.S. National Institutes of Health http://www.clinicaltrials.gov/

[B16] Singer NG Caplan AI Mesenchymal stem cells: mechanisms of inflammation Annu Rev Pathol 2011 6 457 478 10.1146/annurev-pathol-011110-130230 21073342

[B17] Krampera M Galipeau J Shi Y Tarte K Sensebe L; MSC Committee of the International Society for Cellular Therapy (ISCT): **Immunological characterization of multipotent mesenchymal stromal cells - The International Society for Cellular Therapy (ISCT) working proposal** Cytotherapy 2013 15 1054 1061 10.1016/j.jcyt.2013.02.010 23602578

[B18] Lee RH Seo MJ Reger RL Spees JL Pulin AA Olson SD Prockop DJ Multipotent stromal cells from human marrow home to and promote repair of pancreatic islets and renal glomeruli in diabetic NOD/scid mice Proc Natl Acad Sci U S A 2006 103 17438 17443 10.1073/pnas.0608249103 17088535 PMC1634835

[B19] Li J Ezzelarab MB Cooper DK Do mesenchymal stem cells function across species barriers? Relevance for xenotransplantation Xenotransplantation 2012 19 273 285 10.1111/xen.12000 22978461 PMC3445044

[B20] Haskins K Portas M Bradley B Wegmann D Lafferty K T-lymphocyte clone specific for pancreatic islet antigen Diabetes 1988 37 1444 1448 10.2337/diab.37.10.1444 2458291

[B21] Lieberman SM Evans AM Han B Takaki T Vinnitskaya Y Caldwell JA Serreze DV Shabanowitz J Hunt DF Nathenson SG Santamaria P DiLorenzo TP Identification of the beta cell antigen targeted by a prevalent population of pathogenic CD8+ T cells in autoimmune diabetes Proc Natl Acad Sci U S A 2003 100 8384 8388 10.1073/pnas.0932778100 12815107 PMC166238

[B22] Lo Surdo J Bauer SR Quantitative approaches to detect donor and passage differences in adipogenic potential and clonogenicity in human bone marrow-derived mesenchymal stem cells Tissue Eng Part C Methods 2012 18 877 889 10.1089/ten.tec.2011.0736 22563812 PMC3483050

[B23] Gronthos S Graves SE Ohta S Simmons PJ The STRO-1+ fraction of adult human bone marrow contains the osteogenic precursors Blood 1994 84 4164 4173 7994030

[B24] Zheng ZH Li XY Ding J Jia JF Zhu P Allogeneic mesenchymal stem cell and mesenchymal stem cell-differentiated chondrocyte suppress the responses of type II collagen-reactive T cells in rheumatoid arthritis Rheumatology (Oxford) 2008 47 22 30 10.1093/rheumatology/kem284 18077486

[B25] Corcione A Benvenuto F Ferretti E Giunti D Cappiello V Cazzanti F Risso M Gualandi F Mancardi GL Pistoia V Uccelli A Human mesenchymal stem cells modulate B-cell functions Blood 2006 107 367 372 10.1182/blood-2005-07-2657 16141348

[B26] Bai L Lennon DP Eaton V Maier K Caplan AI Miller SD Miller RH Human bone marrow-derived mesenchymal stem cells induce Th2-polarized immune response and promote endogenous repair in animal models of multiple sclerosis Glia 2009 57 1192 1203 10.1002/glia.20841 19191336 PMC2706928

[B27] Abumaree M Al Jumah M Pace RA Kalionis B Immunosuppressive properties of mesenchymal stem cells Stem Cell Rev 2012 8 375 392 10.1007/s12015-011-9312-0 21892603

[B28] Wada N Bartold PM Gronthos S Human foreskin fibroblasts exert immunomodulatory properties by a different mechanism to bone marrow stromal/stem cells Stem Cells Dev 2011 20 647 659 10.1089/scd.2010.0246 20712449

[B29] Zhao S Wehner R Bornhauser M Wassmuth R Bachmann M Schmitz M Immunomodulatory properties of mesenchymal stromal cells and their therapeutic consequences for immune-mediated disorders Stem Cells Dev 2010 19 607 614 10.1089/scd.2009.0345 19824807

[B30] Krampera M Cosmi L Angeli R Pasini A Liotta F Andreini A Santarlasci V Mazzinghi B Pizzolo G Vinante F Romagnani P Maggi E Romagnani S Annunziato F Role for interferon-gamma in the immunomodulatory activity of human bone marrow mesenchymal stem cells Stem Cells 2006 24 386 398 10.1634/stemcells.2005-0008 16123384

[B31] Berman DM Willman MA Han D Kleiner G Kenyon NM Cabrera O Karl JA Wiseman RW O'Connor DH Bartholomew AM Kenyon NS Mesenchymal stem cells enhance allogeneic islet engraftment in nonhuman primates Diabetes 2010 59 2558 2568 10.2337/db10-0136 20622174 PMC3279532

[B32] Baron F Lechanteur C Willems E Bruck F Baudoux E Seidel L Vanbellinghen JF Hafraoui K Lejeune M Gothot A Fillet G Beguin Y Cotransplantation of mesenchymal stem cells might prevent death from graft-versus-host disease (GVHD) without abrogating graft-versus-tumor effects after HLA-mismatched allogeneic transplantation following nonmyeloablative conditioning Biol Blood Marrow Transplant 2010 16 838 847 10.1016/j.bbmt.2010.01.011 20109568

[B33] Toubai T Paczesny S Shono Y Tanaka J Lowler KP Malter CT Kasai M Imamura M Mesenchymal stem cells for treatment and prevention of graft-versus-host disease after allogeneic hematopoietic cell transplantation Curr Stem Cell Res Ther 2009 4 252 259 10.2174/157488809789649269 19500066

[B34] Zhou K Zhang H Jin O Feng X Yao G Hou Y Sun L Transplantation of human bone marrow mesenchymal stem cell ameliorates the autoimmune pathogenesis in MRL/lpr mice Cell Mol Immunol 2008 5 417 424 10.1038/cmi.2008.52 19118507 PMC4072411

[B35] Omori Y Honmou O Harada K Suzuki J Houkin K Kocsis JD Optimization of a therapeutic protocol for intravenous injection of human mesenchymal stem cells after cerebral ischemia in adult rats Brain Res 2008 1236 30 38 18722359 10.1016/j.brainres.2008.07.116 PMC2605387

[B36] Onda T Honmou O Harada K Houkin K Hamada H Kocsis JD Therapeutic benefits by human mesenchymal stem cells (hMSCs) and Ang-1 gene-modified hMSCs after cerebral ischemia J Cereb Blood Flow Metab 2008 28 329 340 10.1038/sj.jcbfm.9600527 17637706 PMC2605394

[B37] Murphy E Shibuya K Hosken N Openshaw P Maino V Davis K Murphy K O'Garra A Reversibility of T helper 1 and 2 populations is lost after long-term stimulation J Exp Med 1996 183 901 913 10.1084/jem.183.3.901 8642294 PMC2192360

[B38] Feili-Hariri M Falkner DH Morel PA Polarization of naive T cells into Th1 or Th2 by distinct cytokine-driven murine dendritic cell populations: implications for immunotherapy J Leukoc Biol 2005 78 656 664 10.1189/jlb.1104631 15961574

[B39] Scheinman EJ Avni O Transcriptional regulation of GATA3 in T helper cells by the integrated activities of transcription factors downstream of the interleukin-4 receptor and T cell receptor J Biol Chem 2009 284 3037 3048 19056736 10.1074/jbc.M807302200

[B40] Hernandez-Hoyos G Anderson MK Wang C Rothenberg EV Alberola-Ila J GATA-3 expression is controlled by TCR signals and regulates CD4/CD8 differentiation Immunity 2003 19 83 94 10.1016/S1074-7613(03)00176-6 12871641

[B41] Augello A Tasso R Negrini SM Amateis A Indiveri F Cancedda R Pennesi G Bone marrow mesenchymal progenitor cells inhibit lymphocyte proliferation by activation of the programmed death 1 pathway Eur J Immunol 2005 35 1482 1490 10.1002/eji.200425405 15827960

[B42] Meisel R Zibert A Laryea M Gobel U Daubener W Dilloo D Human bone marrow stromal cells inhibit allogeneic T-cell responses by indoleamine 2,3-dioxygenase-mediated tryptophan degradation Blood 2004 103 4619 4621 10.1182/blood-2003-11-3909 15001472

[B43] Aggarwal S Pittenger MF Human mesenchymal stem cells modulate allogeneic immune cell responses Blood 2005 105 1815 1822 10.1182/blood-2004-04-1559 15494428

[B44] Sato K Ozaki K Oh I Meguro A Hatanaka K Nagai T Muroi K Ozawa K Nitric oxide plays a critical role in suppression of T-cell proliferation by mesenchymal stem cells Blood 2007 109 228 234 10.1182/blood-2006-02-002246 16985180

[B45] Chabannes D Hill M Merieau E Rossignol J Brion R Soulillou JP Anegon I Cuturi MC A role for heme oxygenase-1 in the immunosuppressive effect of adult rat and human mesenchymal stem cells Blood 2007 110 3691 3694 10.1182/blood-2007-02-075481 17684157

[B46] Gieseke F Schutt B Viebahn S Koscielniak E Friedrich W Handgretinger R Muller I Human multipotent mesenchymal stromal cells inhibit proliferation of PBMCs independently of IFNgammaR1 signaling and IDO expression Blood 2007 110 2197 2200 10.1182/blood-2007-04-083162 17522338

[B47] Plumas J Chaperot L Richard MJ Molens JP Bensa JC Favrot MC Mesenchymal stem cells induce apoptosis of activated T cells Leukemia 2005 19 1597 1604 10.1038/sj.leu.2403871 16049516

[B48] Chang CJ Yen ML Chen YC Chien CC Huang HI Bai CH Yen BL Placenta-derived multipotent cells exhibit immunosuppressive properties that are enhanced in the presence of interferon-gamma Stem Cells 2006 24 2466 2477 10.1634/stemcells.2006-0071 17071860

[B49] Fiorina P Jurewicz M Augello A Vergani A Dada S La Rosa S Selig M Godwin J Law K Placidi C Smith RN Capella C Rodig S Adra CN Atkinson M Sayegh MH Abdi R Immunomodulatory function of bone marrow-derived mesenchymal stem cells in experimental autoimmune type 1 diabetes J Immunol 2009 183 993 1004 10.4049/jimmunol.0900803 19561093 PMC3895445

