# Supplementary figures and images for: Assessment of immunosuppressive activity of human mesenchymal stem cells using murine antigen specific CD4 and CD8 T cells in vitro
Source: Stem Cell Res Ther. 2013 Oct 22;4(5):128. doi: 10.1186/scrt339 (PMC3854780; doi:10.1186/scrt339)

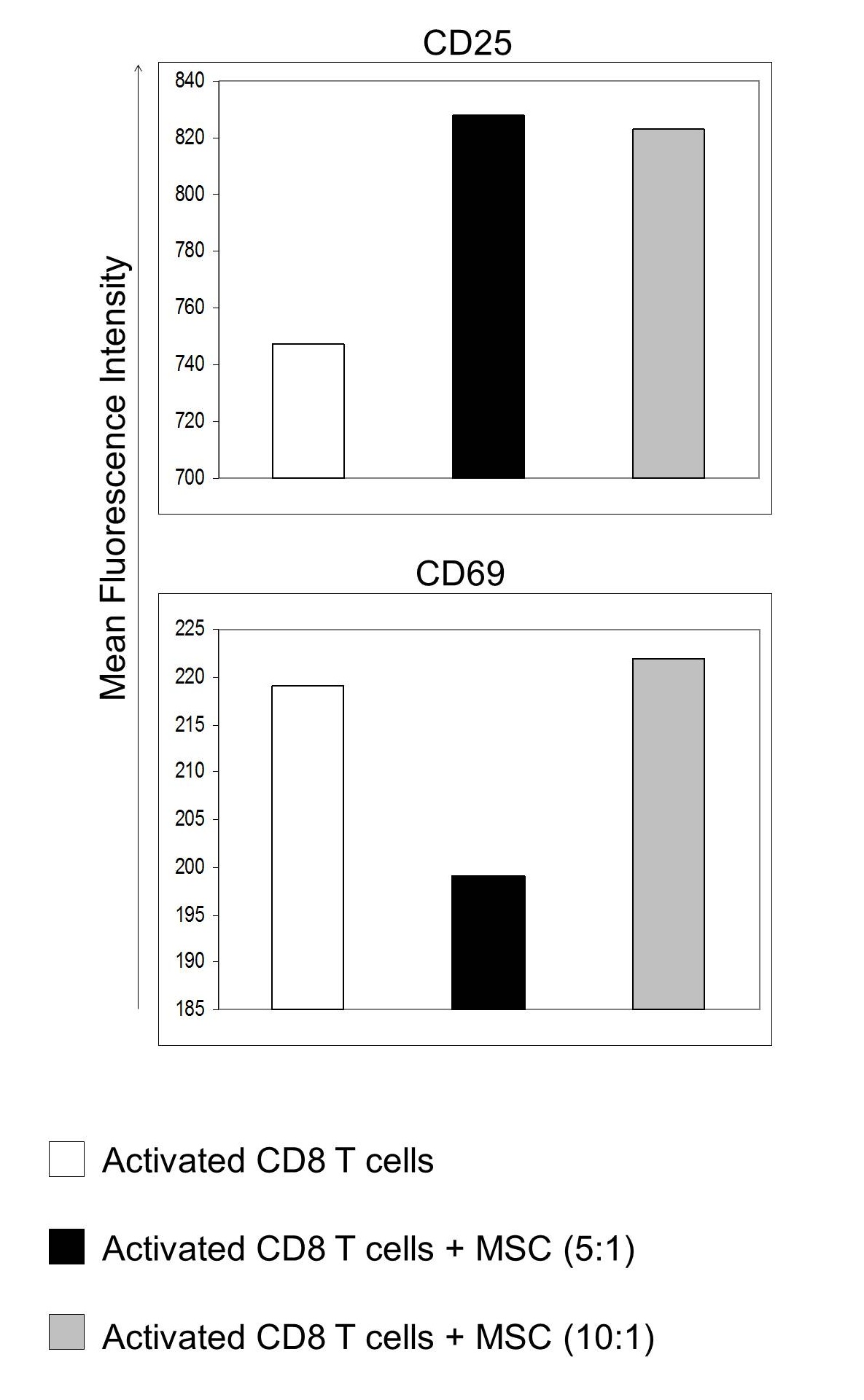

Supplement: Additional file 1: Figure S1 — hMSCs do not affect surface markers on already activated T cells. Mouse CD8+ Ag specific T cells (8.3 T cells) were stimulated with anti-CD3/CD28 Dynabeads (Invitrogen) (1:1 ratio of T cells: beads) and human IL-2 (2,000 U/ml) for three days. Three days later, beads were removed, T cells were then re-stimulated with IGRP peptide-pulsed irradiated spleen cells, and human MSC were added to the culture at two different ratios: 10:1 and 5:1 and further incubated for an additional three days. Activation markers CD25 and CD69 for mouse 8.3 T cells were evaluated and the mean fluorescence intensity was plotted for each condition. The values are representative of three different experiments. [file scrt339-S1.jpeg]

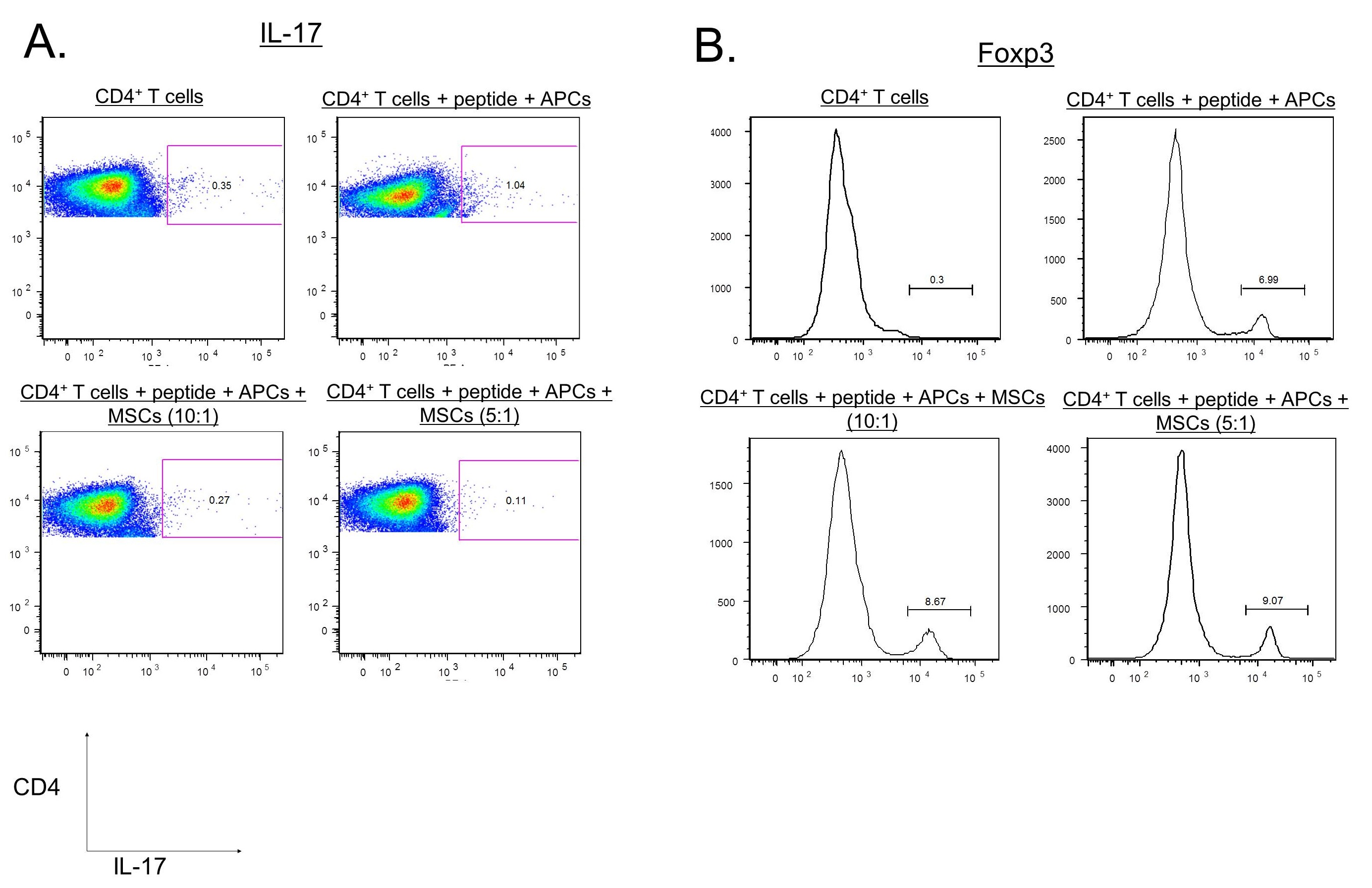

Supplement: Additional file 2: Figure S2 — The presence of hMSCs does not lead to significant changes of Th17/Treg subsets. Purified mouse CD4+ Ag specific T cells (BDC2.5 T cells) were cultured in the presence of Antigen Presenting Cells and their cognate peptide for three days. hMSC were co-cultured with the mouse T cells at a ratio of 5:1 T cells: MSC. For measuring Th17 cells, T cells were stimulated with PMA (50 ng/ml) and Ionomycin (1 μg/ml) in the presence of Golgi-Plug at 37°C for five hours. Then surface staining with anti-CD4 and anti-TCR Vβ4 Abs, permeabilization/fixation (using BD Cytofix/Cytoperm kit) and Intracellular staining for mouse Foxp3 (eFluor450) and IL-17A (PE) was performed according to the manufacture’s instruction (BD). The histograms are representative of two separate experiments. [file scrt339-S2.jpeg]
